# Immunoinformatics and molecular modeling approaches to design a multi-epitope vaccine against Tibrovirus Congo

**DOI:** 10.1515/med-2026-1388

**Published:** 2026-04-08

**Authors:** Muhammad Naveed, Muhammad Asim, Tariq Aziz, Sonia Amjad, Parveen Qadir, Hafiz Muzzammel Rehman, Ayaz Ali Khan, Manal F. Elkhadragy, Ashwag Shami, Maher S. Alwethaynani, Fakhria A. Al-Joufi, Deema Fallatah

**Affiliations:** Department of Biotechnology, Faculty of Science and Technology, University of Central Punjab, Lahore, Pakistan; Laboratory of Animal Health, Hygiene and Food Quality University of Ioannina Arta, Ioannina, Greece; School of Biochemistry & Biotechnology, University of the Punjab, Lahore, Pakistan; Department of Biotechnology, University of Malakand Chakdara Dir Lower, Chakdara, Pakistan; Department of Biology, College of Science, Princess Nourah bint Abdulrahman University, Riyadh, Saudi Arabia; Department of Clinical Laboratory Sciences, College of Applied Medical Sciences, Shaqra University, Alquwayiyah, Riyadh, Saudi Arabia; Department of Pharmacology, College of Pharmacy, Jouf University, Sakaka, Saudi Arabia; Department of Medical Laboratory Sciences, College of Applied Medical Sciences, Prince Sattam Bin Abdulaziz University, Al-Kharj, Saudi Arabia; Department of Microbiology, Faculty of Science and Technology, University of Central Punjab, Lahore, Pakistan

**Keywords:** Tibrovirus Congo, multi-epitope vaccine, molecular docking, molecular dynamics simulations, toll-like receptors, immune simulation

## Abstract

**Objectives:**

The objective of this study was to design and evaluate a computationally optimized multi-epitope vaccine targeting the G protein and nucleoprotein of Tibrovirus Congo using immunoinformatics and molecular simulation approaches.

**Methods:**

B-cell and T-cell epitopes were predicted, screened for antigenicity, allergenicity, and toxicity, and linked with GPGPG, AAY, and KK linkers. A β-defensin-3 adjuvant and a PADRE sequence were incorporated, generating a 224-amino acid vaccine construct. Population coverage was analyzed globally. The tertiary structure was modeled, refined, and validated using Ramachandran plot analysis and ERRAT scoring. Molecular docking was performed with TLR2 and TLR4 receptors, followed by 100 ns molecular dynamics (MD) simulations. Root mean square deviation (RMSD), root mean square fluctuations (RMSF),radius of gyration, dynamic cross-correlation matrix (DCCM), principal component analysis (PCA), and free energy landscape (FEL) analyses were applied to assess stability and motion.

**Results:**

The vaccine construct was predicted to be highly antigenic, non-allergenic, and stable, with 91.81 % global population coverage, highest in Europe and North America. The refined 3D model achieved a Ramachandran favored-region score of 96.3 % and an ERRAT score of 99.63. Docking revealed strong affinity toward TLR2 (−1,665.5, 16 hydrogen bonds) compared to TLR4 (−1,227.8). MD simulations confirmed stable binding, with an average RMSD of 6 Å, a radius of gyration of around 19.8 Å, and RMSF between 1 and 4 Å. DCCM and PCA indicated positive residue correlations and stable conformational dynamics. Immune simulations predicted robust humoral and cellular responses, including elevated IgG, IgM, IL-2, and IFN-γ levels, and long-term memory cell formation.

**Conclusions:**

The designed vaccine construct demonstrates promising immunogenic and structural properties, suggesting potential efficacy against Tibrovirus Congo. Further *in vitro* and *in vivo* validation is required to confirm safety and effectiveness.

## Introduction

Tibrovirus Congo, also known as Bas-Congo virus (BASV), is classified as an emergent virus of the Rhabdoviridae family, which has single-stranded, enveloped, negative-sense RNA viruses that can infect diverse hosts such as humans, livestock, and wildlife [1]. This virus is known to cause febrile illnesses with symptoms including high fever, myalgia, nausea, vomiting, and, in some cases, neurological symptoms [2]. Tibroviruses are mainly vector-borne viruses with arthropods such as midges and mosquitoes, supporting their expansion in tropical and subtropical areas [3]. Still, limited research has been done on these viruses, so their zoonotic potential is of high concern, and further study is needed to understand their ability to cause diseases. Like other rhabdoviruses, Tibrovirus Congo possesses a similar viral entry, replication, and immune evasion mechanism, making it a difficult pathogen to treat using current therapeutic approaches [4]. The prevalence of Tibrovirus Congo has been reported in multiple areas of the world, with an increase in cases associated with some febrile illnesses [5]. Since 2014, infections have gradually increased in the Central and West African regions, Southeast Asia, and some parts of South America [6]. However, due to the lack of reliable diagnostic tools and comprehensive epidemiological studies, the actual burden of this virus remains vastly underestimated. The detection of antibodies in asymptomatic individuals raises concerns about silent transmission cycles, suggesting that the virus may spread unpredictably in regions with suitable vector populations [7].

Currently, there are no antiviral medications specifically available for Tibrovirus Congo infections. The available treatments include supportive care, including antipyretics, fluids, analgesia, and pain management therapeutics [8]. Some antiviral medications have been used in attempts to inhibit related rhabdoviruses, such as ribavirin or favipiravir, but these have not been tested on Tibrovirus Congo [9]. In addition, the drugs would not be effective because of their severe side effects of liver damage, disorders of the blood system, and congenital disabilities [10]. Passive immunotherapy, including monoclonal antibodies, has performed as well in preclinical models. However, the high cost of treatment, limited accessibility, and the risk of viral mutations leading to immune evasion make these therapeutic options unsuitable for large-scale application [11]. Vaccine development, therefore, remains an urgent priority, particularly in endemic regions that face recurring outbreaks, as no preventive strategy currently exists other than the creation of effective vaccines to mitigate this threat [12].

Tibrovirus Congo, like all other members of the Rhabdoviridae family, possesses multiple virulence factors that contribute to its pathogenesis and its capabilities for immune evasion [13]. The glycoprotein (G) of Tibrovirus Congo is an essential protein responsible for mediating host cell receptor binding and membrane fusion, which are critical steps for viral entry [14]. The matrix (M) protein is an integral component of the virion [15]. The efficiency of Tibrovirus Congo replication in mammalian and insect vectors enhances its ability for cross-species transmission and makes it a difficult pathogen to control. The G protein and nucleoprotein were selected as vaccine targets because they play key roles in the infection and immune recognition processes of Tibrovirus Congo [12]. The G protein mediates viral attachment and membrane fusion, making it a major surface antigen exposed to host immune surveillance, while the nucleoprotein is highly conserved and critical for viral genome encapsulation and replication [5], 16]. Both proteins have been shown in related Rhabdoviridae viruses to elicit strong humoral and cellular immune responses, making them promising targets for multi-epitope vaccine design [17], 18].

To our knowledge, this is the first study to design a multi-epitope vaccine candidate specifically targeting Tibrovirus Congo using an integrated immunoinformatics and molecular modeling approach. Unlike previous studies that focused on common viral pathogens, this work addresses an underexplored emerging virus with significant zoonotic potential, thus contributing new insights into computational vaccine design for neglected viral families. The virulence factors of Tibrovirus Congo necessitate the design of targeted vaccines to prevent and neutralize these infections. This research aims to design a multi-epitope vaccine for Tibrovirus Congo using immunoinformatics approaches [19]. Advanced bioinformatics and immunoinformatics analysis tools characterized highly immunogenic B-cell and T-cell epitopes. Predicted epitopes undergo thorough evaluation for antigenicity, allergenicity, and toxicity to determine their feasibility for vaccine development. A multi-epitope vaccine construct, featuring selected epitopes, adjuvants, and linker sequences to enhance immunogenicity, was developed. The final construct integrates selected epitopes with appropriate adjuvants and linkers to enhance overall immunogenicity. This is followed by molecular docking, molecular dynamics simulations, and immune simulations to evaluate its interaction stability and predicted immune response.

## Materials and methods

### Retrieval of target proteins

The target proteins of Tibrovirus Congo were obtained from the UniProt database (https://www.uniprot.org/uniprotkb) [20], a well-known database for the description and annotation of protein sequences. The selected proteins were obtained in FASTA format for further analysis.

### Epitope prediction

The Bepipred Linear Epitope Prediction 2.0 from IEDB tool (http://tools.iedb.org/bcell/) was used to predict linear B-cell epitopes from the protein sequences, with a cutoff value of 0.5 for regions estimated to elicit a humoral immune response and bind to antibodies [21]. MHC Class I-restricted epitopes were predicted using the ANN 4.0 tool (http://tools.iedb.org/mhci/), which assesses the binding of peptides to MHC Class I complexes to measure the potential for activating cytotoxic T lymphocyte responses [22]. For MHC Class II-restricted epitopes, the NetMHCIIpan 2.3 (NN-align 2.3) tool (http://tools.iedb.org/mhcii/) was used to predict which peptides can bind to MHC Class II, necessary for the activation of T helper cells [23].

### Epitope screening

The antigenic properties of the predicted epitopes were evaluated using the VaxiJen 2.0 server (https://www.ddg-pharmfac.net/vaxijen/VaxiJen/VaxiJen.html) with a cutoff value of 0.5 to differentiate antigenic epitopes from nonantigenic. Potential allergenic risks associated with epitopes were predicted using the AllerTOP v2.1 server (https://www.ddg-pharmfac.net/allertop_test/). The toxicity of the epitopes was evaluated using ToxinPred v3.0 (https://webs.iiitd.edu.in/raghava/toxinpred3/). This extensive screening enabled the selection of non-toxic, highly immunogenic epitopes to design a multi-epitope vaccine construct against Tibrovirus Congo [24].

### Population coverage analysis

Population coverage analysis was performed using the IEDB Population Coverage Analysis tool (http://tools.iedb.org/population/). This tool estimated global and regional immune response potential based on the predicted binding affinities of selected epitopes to various MHC alleles across different ethnic populations. By integrating allele frequency data with epitope–HLA binding profiles, the analysis predicted the proportion of individuals likely to mount an effective immune response [25].

### Vaccine construct design

The multi-epitope vaccine design incorporated B-cell, MHC Class I, and MHC Class II epitopes. These epitopes were joined with AAY, GPGPG, and KK linkers, which enhance the folding and presentation of epitopes in the vaccine construct [26]. Additionally, a β-defensin-3 adjuvant was incorporated at the N-terminal of the construct to improve immune response through activation of innate immunity [27]. Moreover, to further increase T-cell activation, an adjuvant in the form of a well-known T-helper epitope called PADRE sequence was incorporated to enhance T-cell-driven responses. A 6× His tag was introduced at the C-terminal of the construct to increase the ease of purification and detection of the recombinant protein during experimental validation [28]. The antigenic potential of the vaccine construct was predicted using VaxiJen v2.0, non-allergenicity was evaluated by AllerTOP v2.1, and toxicity assessment was done using ToxinPred v3.0.

### Physicochemical properties

The physicochemical features of the multi-epitope vaccine construct were assessed with the ExPASy ProtParam tool (https://web.expasy.org/protparam/) [29]. The tool provides detailed information on molecular weight, amino acid composition, isoelectric point, extinction coefficient, aliphatic index, and stability index of the protein. The additional solubility of the vaccine construct was analyzed using SoluProt v1.0 (https://loschmidt.chemi.muni.cz/soluprot/) with a cutoff value of 0.5 [30].

### Secondary structure prediction

The PSIPRED (http://bioinf.cs.ucl.ac.uk/psipred/) and GOR IV(https://npsa-prabi.ibcp.fr/cgi-bin/npsa_automat.pl?page=/NPSA/npsa_gor4.html) tools were used to predict the secondary structures of the multi-epitope vaccine [31], 32]. PSIPRED is one of the most widely used tools that increases prediction accuracy by using neural networks to predict α-helices, β-strands, and coil regions from sequence information [33]. GOR IV is also a reliable method that uses a statistic-based technique to formulate an underlying protein secondary structure prediction framework. Both tools were used to predict the structural aspects of the vaccine construct to ensure that the epitopes are optimally presented for immune system recognition [34].

### Tertiary structure prediction

The tertiary structure of the multi-epitope vaccine construct was predicted using the advanced deep learning-based tool AlphaFold3 (https://alphafoldserver.com/), which provides precise predictions of protein structures. AlphaFold3 predicts the 3D conformation of a protein from the sequence of its amino acids while considering the interactions within the protein that dictate its structure [35], 36]. Subsequently, this model was further improved with GalaxyRefine (https://galaxy.seoklab.org/cgi-bin/submit.cgi?type=REFINE). This tool enhances model accuracy by applying iterative molecular dynamics simulations to optimize the protein conformation, a process termed model refinement. The protein structure geometry verification was done using the Ramachandran plot (https://saves.mbi.ucla.edu/), which ensures that the φ and ψ dihedral angles are in favorable conformations [37]. The overall structural quality of the vaccine model was further evaluated using the ERRAT tool (https://www.doe-mbi.ucla.edu/errat/), which analyzes non-bonded atomic interactions and identifies regions with potential steric clashes [38].

### Discontinuous B-cell epitope prediction

The ElliPro server (http://tools.iedb.org/ellipro/) was used to predict discontinuous B-cell epitopes of the multi-epitope vaccine construct with a threshold score of 0.5 for epitope selection. Ellipro predicts conformational epitopes based on 3D models of proteins by selecting portions that are usually at the surface and can bind with antibodies. For residue clustering, the maximum distance was set at 6 Å so that the residues selected for the epitope are close to one another in the protein tertiary structure, regardless of how far apart the residues are in the primary sequence [39].

### Disulfide engineering

To increase the structural stability of the designed vaccine, disulfide bond engineering was performed with Disulfide by Design 2 v2.13 (DbD2) (http://cptweb.cpt.wayne.edu/DbD2/). This tool predicted potential residue pairs for disulfide bond formation by examining the Cα-Cβ-Sγ bond angle and the χ3 dihedral angle, where covalent connection formation occurs. Analysis was done using the software’s default parameters, where the χ3 angle was set at −87° or +97° (± 30°) and the Cα-Cβ-Sγ bond angle was held at 114.6° ± 10° [40].

### Molecular docking and interaction analysis

The designed vaccine construct was docked with TLR2 and TLR4 to analyze its potential to bind with the immune receptors that help trigger an innate immune response. Docking was performed using ClusPro 2.0 (https://cluspro.bu.edu/login.php), a protein-protein docking tool that generates various models based to binding energy and cluster rankings to identify the most stable and biologically relevant complexes [41]. Discovery Studio v24.1.0 was used for the visualization of docked complexes, whereas PDBsum (https://www.ebi.ac.uk/thornton-srv/software/PDBsum1/) was used for detailed analysis of molecular interactions, including hydrogen bonds, hydrophobic contacts, and salt bridges between vaccine and TLRs [42].

### Molecular dynamics simulations

Molecular dynamics (MD) simulation was conducted with Desmond (D.E. Shaw Research) to analyze the interaction stability of the vaccine-TLR2 complex. The initial steps of MD simulation involved preparing the TLR2 receptor and vaccine complex through multiple optimization and minimization steps, completed using System Builder tools [43]. The system was neutralized by sodium and chloride ions, and a 0.15 M NaCl buffer was added to model physiological conditions. The NPT ensemble was used for the system energy minimization. The simulations proceeded after a relaxation step for 100 ns at 300 K and 1 atm pressure, using the OPLS4 force field. The particle mesh Ewald method calculated electrostatic interactions with a cut-off for Coulomb interactions of 9.0 Å [44]. The temperature control was done with a Nosé–Hoover chain thermostat, while pressure was maintained with a Martyna-Tuckerman-Klein chain piston at a 2.0 ps coupling constant. The Root mean square deviation (RMSD) and Root mean square fluctuations (RMSF) values were monitored over time to determine the system’s stability. The trajectory data was collected every 100 ps. The radius of gyration (Rg) was computed using GROMACS. The Desmond trajectory files were converted to XTC format with MDTraj and analyzed using the gmx gyrate command [45].

#### Principal component analysis

Principal component analysis is one of the most common methods for analyzing the trajectories of MD simulations by reducing the high-dimensional data into lower dimensions. This was done by mapping the atomic coordinates of interest, in this case, the C-alpha atoms, onto a set of orthogonal vectors. This includes building the covariance matrix of the nuclear coordinates, followed by diagonalization of the matrix to yield its eigenvectors and eigenvalues. The generated eigenvectors are the principal components that capture the given system’s dominant motion patterns. In this study, GROMACS was used to run PCA computations. Desmond simulation trajectories were first converted into the XTC format using MDTraj. The covariance matrix was calculated and diagonalized with the gmx cover command to produce principal components. Also, to understand conformational changes within the system, the gmx anaeig command with the -proj option was used to project the trajectory onto the principal components [46].

#### Dynamic cross-correlation matrix (DCCM)

A dynamic cross-correlation matrix was used to analyze the correlation in the motions of residues within a protein. These motions can have a positive correlation, negative correlation, anticorrelated, or uncorrelated, creating a network of interactions [43]. For this purpose, C-alpha atoms were used in the study to represent residue movements. In contrast, the correlation between residues was calculated based on the cross-correlation coefficient C (i, j), defined as follows:

$$C\;\left(i,j\right)=\frac{<\Delta r_{i}\;.\;\Delta r_{j}\;>}{<{\left|\Delta r_{i}\right|}^{2}{\;>}^{\frac{1}{2}}\;<\;{\left|\Delta r_{j}\right|}^{2}{>}^{\frac{1}{2}}}$$

In this equation, $\Delta r_{i}$ is the displacement vector of the C-alpha atom of one residue is considered. C (i, j) >0 represents correlation, C (i, j) <0 represents anti-correlation, while C (i, j)=0 represents no correlation [47].

MDTraj was used to convert the Desmond simulation trajectories from the DCD format. Then, DCCM calculations were done using the Bio3D package (R).

#### Free energy landscape (FEL)

The free energy landscape (FEL) was used to analyze energy changes during molecular dynamics simulations by relating conformational changes to a set of principal components obtained from principal component analysis [48]. This technique helps identify the energy landscape of the metastable states of the protein under different conditions. In this case, GROMACS was used for FEL analysis. The Gibbs free energy was computed as a function of the first two principal components, PC1 and PC2, using the gmx sham command [49]. To increase the clarity of the FEL plots, locally estimated scatterplot smoothing (LOESS) regression was performed. Also, kernel density estimation (KDE) plots were generated from PCA results to validate the FEL plots [43].

### Immune simulations

The C-ImmSim server (https://kraken.iac.rm.cnr.it/C-IMMSIM/index.php) was used to predict the immune activation capability of the designed vaccine construct. The simulation was conducted with the following parameters: random seed 12345, simulation volume was 10, and 100 steps to analyze the response over time. An adjuvant dose of 100 antigen molecules per injection and 1,000 for the base control group was administered at step 1. The simulation measured the following immune parameters: antigen clearance, cytokine release, and subsequent activation of B and T cells with a memory response [50].

### *In silico* cloning

EMBOSS Backtranseq tool (https://www.ebi.ac.uk/jdispatcher/st/emboss_transeq) was used to reverse the vaccine’s translation into a nucleotide sequence [51]. To increase the translation and expression of the vaccine in the host, codon optimization was done using ExpOptimizer (NovoPro) (https://www.novoprolabs.com/tools/codon-optimization), a codon optimization tool. This optimization ensured the selection of highly preferred codons, enhancing the Codon Adaptation Index (CAI) to increase protein yield and stability [52]. The optimized nucleotide sequence was used for *in silico* cloning and cloned into the pET-28a (+) expression vector using SnapGene v8.0.0 software (https://www.snapgene.com/). This vector was selected because of its higher expression efficiency in bacterial systems, ensuring transcription and translation of the recombinant vaccine construct are done perfectly [53].

### Ethical approval

Not applicable.

## Results

### Retrieval of target proteins

The G protein and nucleoprotein were selected as target proteins for vaccine design against the Tibrovirus Congo. The proteins were retrieved from the UniProt database with UniProt IDs of K0A194 and K0A1N2.

### Selection of B-cell epitopes

Based on the antigenic score, non-allergenicity, and non-toxic profile, one B-cell epitope from each protein, G protein and nucleoprotein of Tibrovirus Congo, was selected for vaccine design, as presented in Table 1. The selected B-cell epitopes were chosen for their high antigenicity, non-allergenicity, and non-toxic nature, ensuring their potential to induce a safe and effective humoral immune response. These epitopes are predicted to be surface-exposed and accessible to B-cell receptors, facilitating recognition and neutralizing antibody production. Including such epitopes is essential for stimulating long-lasting antibody-mediated immunity against Tibrovirus Congo.

**Table 1:** Selected B-cell epitopes.

Protein name	Epitope sequence	Start	End	Antigenicity	Allergenicity	Toxicity
G protein	DFNGTAEEKCDAQHWECFKV	192	211	0.8577	Non-allergen	Non-toxic
Nucleoprotein	VLNPAY	123	128	0.78	Non-allergen	Non-toxic

### Selection of T-cell epitopes

Based on their predicted binding affinity with HLA alleles, multiple epitopes from the G protein and nucleoprotein of Tibrovirus Congo were identified using the IEDB server for both MHC Class I and MHC Class II molecules. The shortlisted epitopes were primarily screened according to their IC_50_ values, as lower IC_50_ values indicate stronger binding affinity with respective HLA alleles. Further selection was refined based on antigenicity, non-allergenicity, and non-toxicity to ensure immunogenic potency and safety. The finalized MHC-I epitopes are presented in Table 2, while the selected MHC-II epitopes are given in Table 3.

**Table 2:** Selected epitopes of MHC Class I.

Protein name	Epitope sequence	Antigenicity	Allergenicity	Toxicity	Alleles
G protein	FCLTAIHAIV	0.9165	Non-allergen	Non-toxic	HLA-A*02:03, HLA-A*02:01
	GIYHRNTSMK	0.9267	Non-allergen	Non-toxic	HLA-A*03:01, HLA-A*11:01
Nucleoprotein	MAFVLLSIY	1.1703	Non-allergen	Non-toxic	HLA-B*35:01, HLA-A*30:02, HLA-B*57:01, HLA-B*58:01
	QIFVKNSRK	0.7429	Non-allergen	Non-toxic	HLA-A*68:02, HLA-A*02:03, HLA-A*02:06, HLA-A*02:01

**Table 3:** Selected epitopes of MHC Class II.

Protein name	Epitope sequence	Antigenicity	Allergenicity	Toxicity	Alleles
G protein	VLCIKIINLIYRFYK	0.8642	Non-allergen	Non-toxic	HLA-DRB1*12:01, HLA-DRB1*15:01, HLA-DRB5*01:01, HLA-DRB1*11:01
	ILRNDVGVSFKDLGF	1.4796	Non-allergen	Non-toxic	HLA-DRB3*01:01, HLA-DRB1*13:02, HLA-DRB1*03:01
Nucleoprotein	LSIAHICKLLGKEIE	0.8553	Non-allergen	Non-toxic	HLA-DRB1*11:01, HLA-DRB1*01:01, HLA-DRB1*07:01
	KGLGLVTRSIYSASA	0.967	Non-allergen	Non-toxic	HLA-DRB1*01:01, HLA-DRB1*11:01

The predicted T-cell epitopes were selected based on their strong binding affinity with multiple HLA Class I and Class II alleles, as indicated by low IC_50_ values, reflecting their high potential for presentation by antigen-presenting cells. Epitopes showing high antigenicity, non-allergenicity, and non-toxicity were prioritized to ensure both immunogenicity and safety. This combination of strong HLA binding and favorable immunological properties increases the likelihood of broad population coverage and effective activation of helper (CD4^+^) and cytotoxic (CD8^+^) T-cell responses, making them promising candidates for inclusion in the final multi-epitope vaccine construct.

### Vaccine construct design

**Figure 1:**
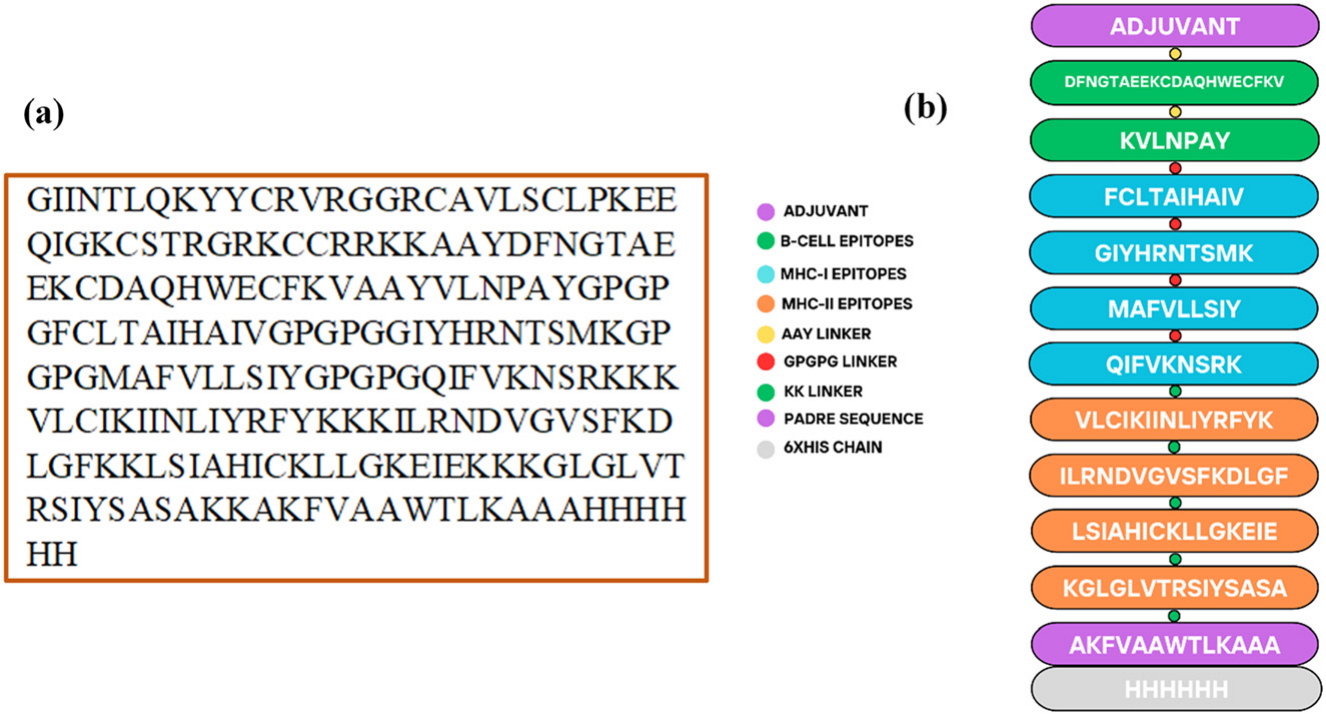
Designed vaccine construct. (a) FASTA sequence of the vaccine construct. (b) Schematic representation showing the arrangement of epitopes and linkers in the construct.

The multi-epitope vaccine construct was designed by integrating several immunological components, including a β-defensin-3 adjuvant, AAY, GPGPG, and KK linkers, a PADRE sequence, and selected B-cell and T-cell epitopes (Figure 1a). The β-defensin-3 adjuvant was incorporated at the N-terminal to enhance immune activation. One B-cell epitope from each of the G protein and nucleoprotein was linked using AAY linkers to ensure proper folding and antigen presentation. The selected four MHC-I and four MHC-II epitopes from both proteins were included to induce robust cytotoxic and helper T-cell responses, connected with GPGPG and KK linkers to facilitate efficient antigen processing and presentation. A PADRE sequence was added toward the C-terminal to improve immune recognition, and a 6× His tag was incorporated for purification purposes (Figure 1b). The final construct contained 224 amino acids and was predicted to be antigenic with a predicted antigenicity score of 0.5739 by VaxiJen v2.0, non-allergenic by AllerTOP v2.1, and non-toxic by ToxinPred.

### Population coverage analysis

**Figure 2:**
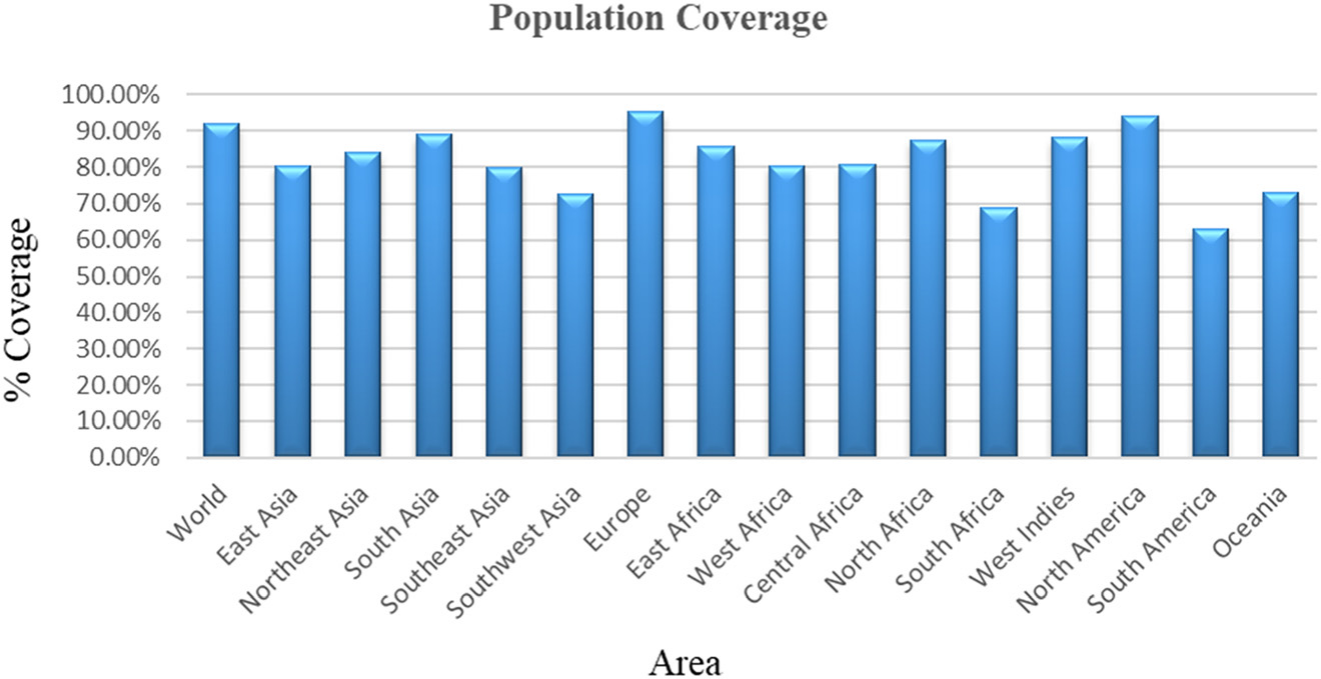
Graphical representation of predicted population coverage of the vaccine construct across global regions.

The population coverage analysis of the T-cell epitopes selected for the Tibrovirus Congo vaccine construct showed significant global coverage. The overall population coverage was predicted to be 91.81 % globally, which shows strong immunogenic prospects. Europe (95.22 %) and North America (94.23 %) had the highest coverage, followed by South Asia (88.95 %), the West Indies (88.16 %), and North Africa (87.45 %). Also, East Africa (85.61 %) and Northeast Asia (83.84 %) exhibited considerable population coverage, indicating the possible success of the vaccine in those regions. Central and West Africa, Southeast Asia, and South America had moderate population coverage. Central Africa (80.50 %) and West Africa (80.36 %) provided good coverage alongside Southeast Asia (79.88 %), while the South American region showed the least coverage, 63.15 %. The predicted population coverage in the different areas of the world is given in Figure 2.

### Physicochemical properties

The predicted physicochemical properties of the vaccine construct are given in Table 4, which shows that the vaccine is thermostable, hydrophilic, and, therefore, soluble. The instability index (26.63) categorizes it as stable. Moreover, durable expression is indicated by an estimated half-life of more than 10 h in *E. coli*. The value suggests good solubility of the protein and thus can be used for experimental verifications and formulation of the vaccine. The predicted physicochemical properties of the vaccine construct are given in Table 4.

**Table 4:** Physicochemical properties of the designed vaccine construct.

Physicochemical properties	
Number of amino acids	224
Molecular weight	24,759.34 Da
Theoretical pI	9.98
Total number of negatively charged residues (Asp + Glu)	11
Total number of positively charged residues (Arg + Lys)	41
Total number of atoms	3,535
Aliphatic index	83.66
Grand average of hydropathicity (GRAVY)	−0.203
Instability index	26.63
Estimated half life in *E. coli* (*in vivo*)	>10 h
Solubility	0.876

### Secondary structure prediction

**Figure 3:**
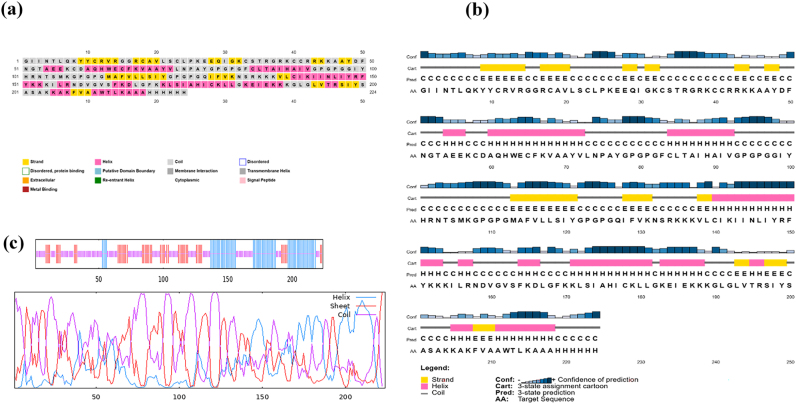
Predicted secondary structure of the vaccine construct. (a) PSIPRED visualization showing helices, coils (grey), and β-strands (yellow). (b) PSIPRED cartoon depicting the predicted secondary structure with confidence scores. (c) GOR IV pie chart showing the structural composition of the construct.

The secondary structure of the vaccine was predicted by PSIPRED and GOR IV, as shown in Figure 3, revealing the composition of secondary elements, including alpha helices, beta strands, and coils. According to PSIPRED, the structure of the vaccine is composed of 74 residues of alpha helices (33.05 %), 41 residues of beta strands (18.30 %), and 109 residues of random coils (48.66 %). Meanwhile, GOR IV showed 64 residues of alpha helices (28.57 %), 53 residues of beta strands (23.66 %), and 107 random coils (47.77 %). There were slight differences in the percentage of secondary units because both methods use different training datasets and algorithms. However, it is notable that both methods showed a proportion of random coils, more significant than the rest, is present in the vaccine protein, along with alpha helices and beta strands. The high proportion of random coils indicates flexibility in the structure, which may facilitate dynamic interactions with immune receptors.

### Tertiary structure prediction

**Figure 4:**
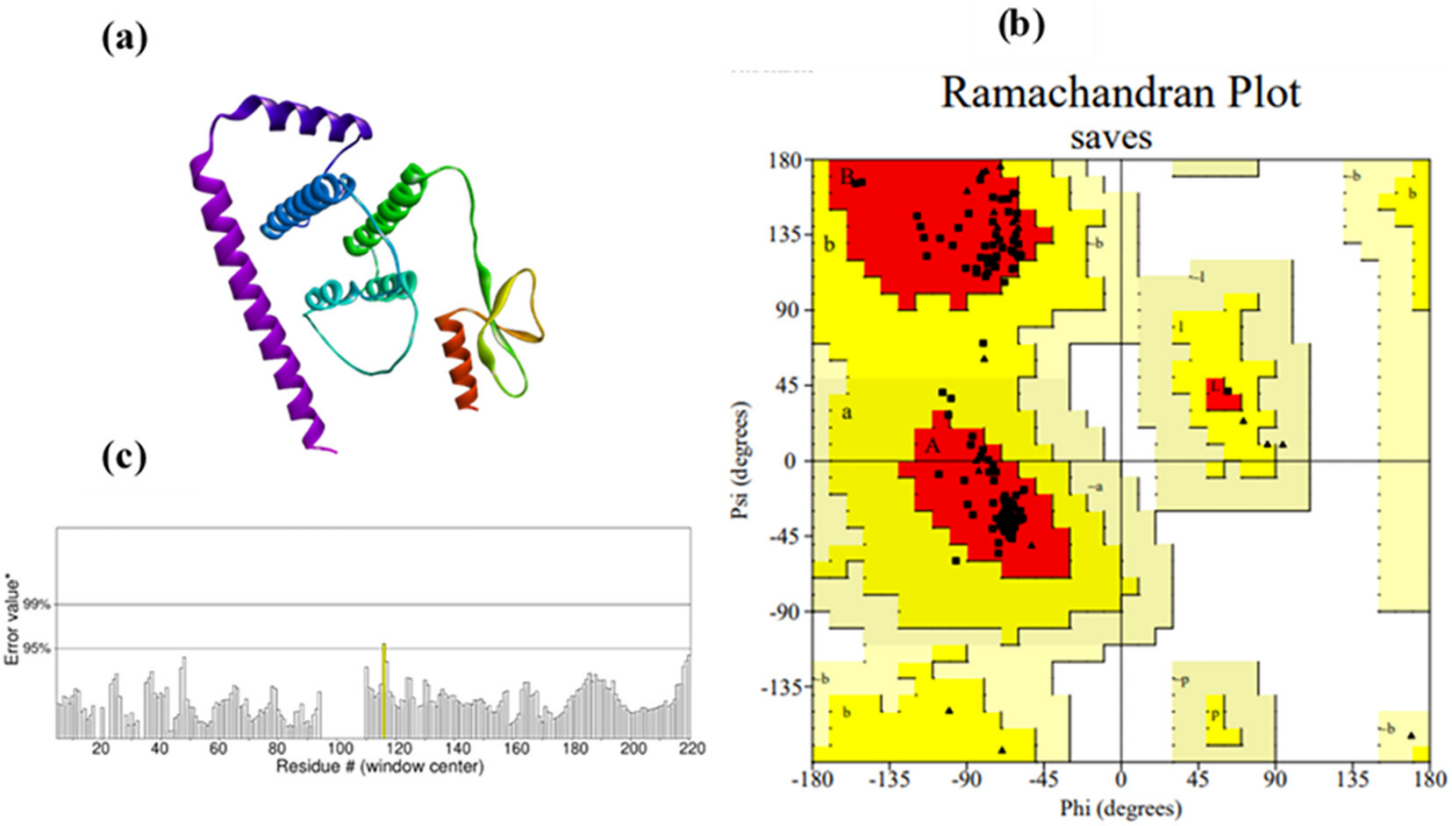
Structural modeling and validation of the designed multi-epitope vaccine construct. (a) Predicted 3D structure of the vaccine protein generated using AlphaFold3. (b) Ramachandran plot showing 97.4 % of residues in favored regions. (c) ERRAT quality assessment plot indicating high structural reliability of the refined model.

The predicted tertiary structure of the vaccine by AlphaFold3 is given in Figure 4a. This structure was refined by GalaxyRefine to improve its quality. Before refinement, Ramachandran plot analysis revealed that 88.4 % of residues were in the most favored regions, 11.6 % were in additionally allowed regions, and no residues were in the generously allowed or disallowed areas. After optimization, the structure showed significant improvement, with 96.3 % of residues in the most favored regions and 3.7 % in the additionally allowed regions, as shown in Figure 4b. This indicates that the structure has improved backbone conformation with no steric clashes and minimal issues. In addition, the ERRAT score of 99.6337 further validates the reliability and accuracy of the tertiary structure of the vaccine, as shown in Figure 4c.

### Discontinuous B-cell epitope prediction

**Figure 5:**
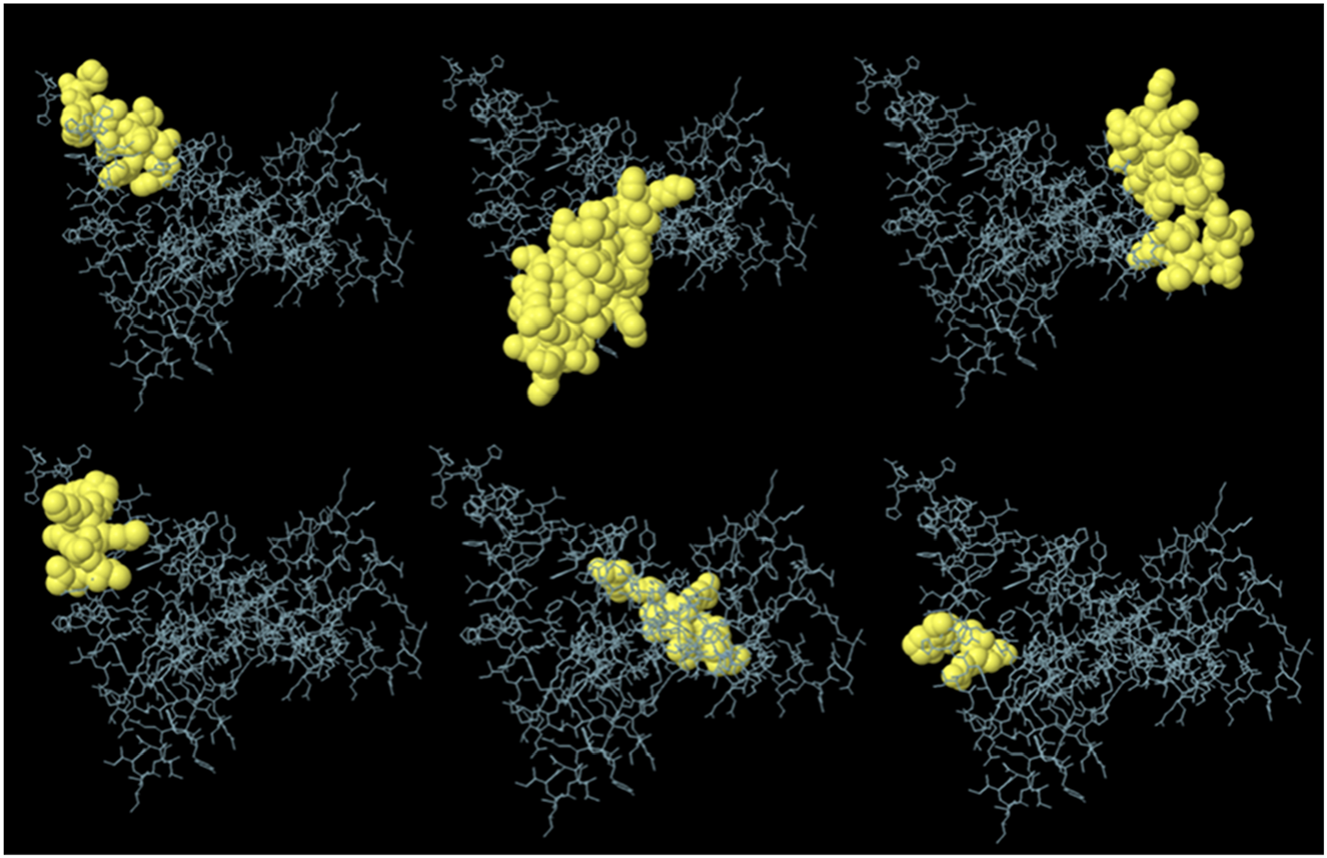
Predicted discontinuous B-cell epitopes shown in yellow color.

The ElliPro server predicted six discontinuous B-cell epitopes with varying immunogenic potential (Figure 5). The highest-ranked epitope (Score: 0.818) contains 13 residues (F208-A221) and is predicted to be the most immunodominant due to its high surface exposure and accessibility for antibody binding. The second epitope (Score: 0.763) includes 35 residues (G1-C41) with glycine and cysteine residues that provide flexibility for effective antibody interaction. The third epitope (Score: 0.701) comprises 24 residues (F150-K183) with mixed hydrophobic and polar amino acids, making it a strong candidate for antibody recognition (Table 5). The remaining epitopes (Scores: 0.662–0.627) contain residues associated with loop or surface regions that enhance antigenicity. Overall, the top ranked epitopes, particularly epitopes 1 and 2, are predicted to play a central role in eliciting a robust humoral immune response.

**Table 5:** Residues of predicted discontinuous B-cell epitopes.

Sr. No	Residues	Number of residues	Score
1	A: F208, A: A210, A: A211, A: W212, A: T213, A: L214, A: K215, A: A216, A: A217, A: A218, A:H219, A:H220, A:H221	13	0.818
2	A:G1, A:I2, A:I3, A:N4, A:T5, A:L6, A:Q7, A:K8, A:C11, A:R12, A:G15, A:G16, A:R17, A:C18, A:A19, A:V20, A:L21, A:S22, A:C23, A:L24, A:P25, A:K26, A:E27, A:Q29, A:G31, A:K32, A:C33, A:S34, A:T35, A:R36, A:G37, A:R38, A:K39, A:C40, A:C41	35	0.763
3	A:F150, A:K154, A:I155, A:L156, A:N158, A:D159, A:V160, A:G161, A:V162, A:S163, A:F164, A:K165, A:D166, A:L167, A:G168, A:F169, A:K170, A:K171, A:L172, A:S173, A:H176, A:K179, A:L180, A:K183	24	0.701
4	A: T104, A: S105, A:M106, A: K107, A: G108, A: P109, A: G110, A: P111, A: G112, A: A114, A: F115, A: L118	12	0.662
5	A: G190, A: L191, A: G192, A: L193, A: V194, A: R196, A: S197, A: S200, A: K204	9	0.627
6	A: G122, A: P123, A: G124, A: P125, A: G126, A: Q127, A: I128	7	0.627

### Disulfide engineering

**Figure 6:**
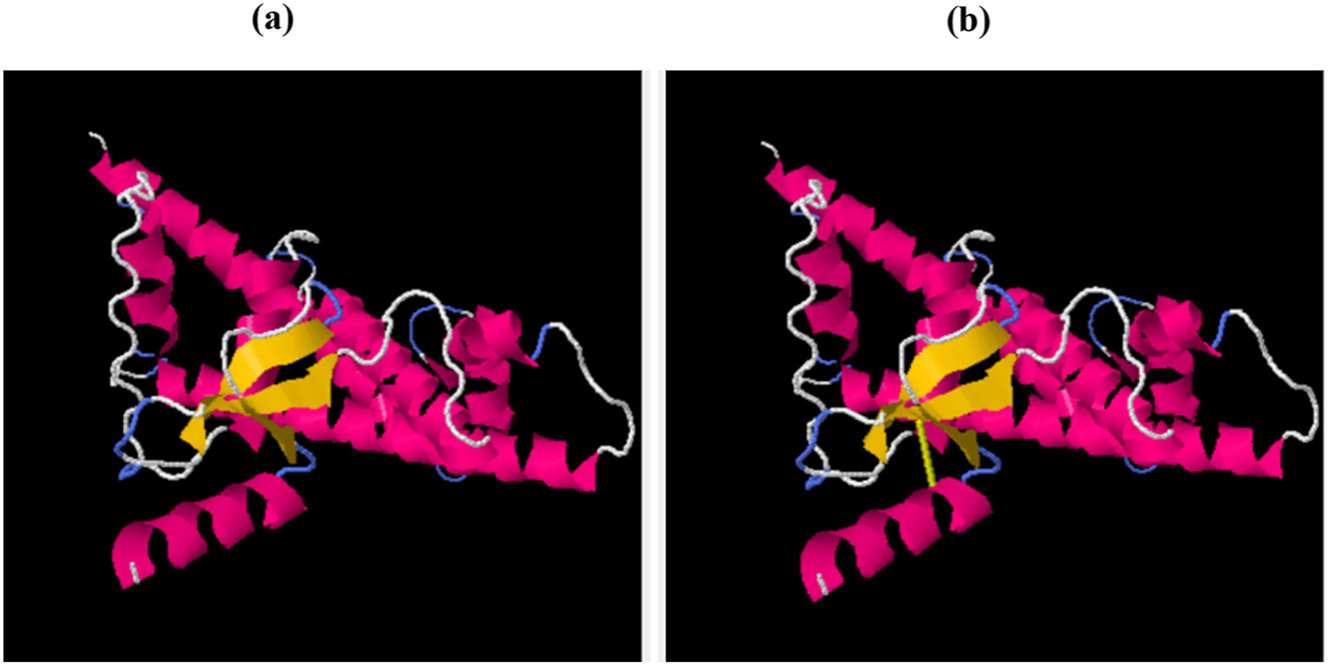
Disulfide engineering of the vaccine construct. (a) Original vaccine structure. (b) Mutated vaccine structure after introduction of disulphide bond as shown with yellow line.

The disulfide bond was introduced in the vaccine construct using Disulfide by Design 2 (DbD2) to increase the structural stability of the designed multi-epitope vaccine. A cysteine pair at positions A:11 and A:40 was selected for bond formation based on optimal geometric parameters for bonding. The analysis predicted a bond energy of +107.71, suggesting a favorable interaction, coupled with a confidence score of 1.00, indicating a highly reliable prediction. A deviation score of 0.0 implies that bond geometry is ideal with no structural distortion. The original structure is represented in Figure 6a, and the mutated vaccine construct in Figure 6b with a disulfide bond introduced between A:11–CYS and A:40–CYS.

### Molecular docking and interaction analysis

The docking results obtained from ClusPro 2.0 showed that the vaccine had a stronger interaction with TLR2, as confirmed by binding energies of −1,665.5, compared to TLR4, which has a binding energy of −1,227.8. Moreover, TLR2 exhibited a more significant number of cluster members, 137, compared to TLR4, which has 93, suggesting an enhanced cluster population and increased probable stability of vaccine binding to TLR2. In addition, the overall number of identified hydrogen bonds in the TLR2 complex, which was 15 compared to 10 in the TLR4 complex, also supports stronger molecular interactions and improved engagement of the TLR2 receptor. The results indicate that although the vaccine can interact with both receptors, greater binding affinity with TLR2 suggests that TLR2 is likely to be more active in modulating immune responses induced by the vaccine (Table 6).

**Table 6:** Docking results of vaccine with TLR2 and TLR4.

Sr. No	Complex	Lowest energy	No. of members	Hydrogen bonds
1	Vaccine-TLR2	−1,665.5	137	16
3	Vaccine-TLR4	−1,227.8	93	10

**Figure 7:**
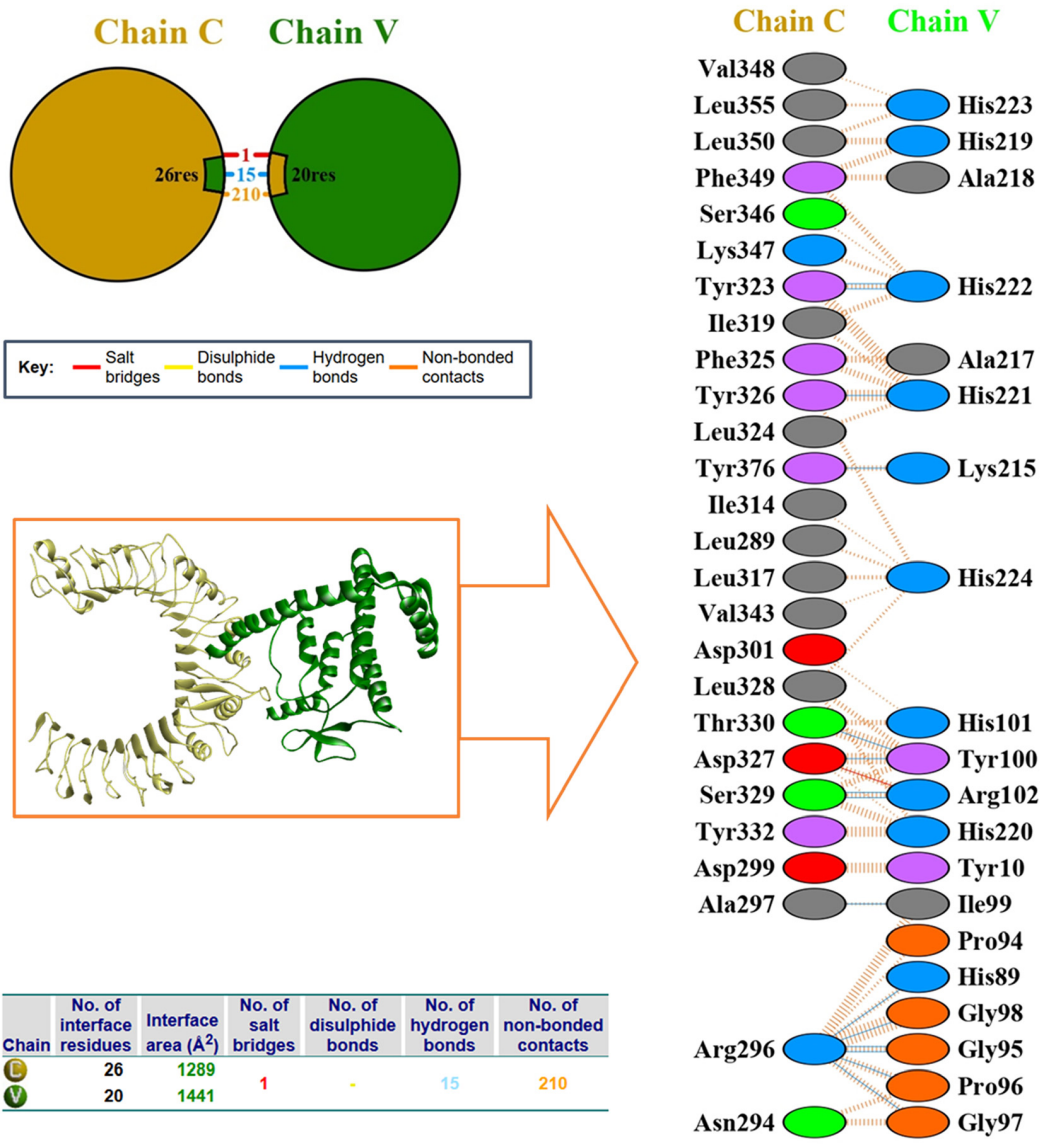
Schematic diagram of interactions between vaccine construct and TLR-2 receptor.

The interaction analysis showed strong molecular interactions between the vaccine and the TLR2 receptor. The interface encompasses 26 residues of TLR2 (Chain C) and 20 residues of the vaccine construct (Chain V), resulting in interface areas of 1,289 Å^2^ for TLR2 and 1,441 Å^2^ for the vaccine, respectively, indicating a large interface area. Remarkably, one salt bridge in the complex increases electrostatic stability and most likely assists in activating the receptor (Table 7). Furthermore, 15 specific hydrogen molecules have strong bonds between the vaccine and TLR2. Moreover, 210 non-bonded contacts strengthen the bonds and suggest a well-defined complex without steric clashes. The docking results indicate that the vaccine interacts firmly and stably with TLR2, forming several hydrogen bonds, non-bonded interactions, and a salt bridge, which enhances its stability on a molecular level. This indicates that TLR2 can effectively recognize and bind the vaccine, potentially triggering strong immune responses (Figure 7).

**Table 7:** Interacting residues of vaccine (chain V) with TLR2 receptor (Chain B).

Sr. No	TLR2 receptor (chain c)	Vaccine (chain V)	Type of bond	Distances (Å)
1	ARG296	GLY95	Hydrogen bond	2.99
2	ARG296	PRO96	Hydrogen bond	2.92
3	ARG296	GLY98	Hydrogen bond	2.87
4	ARG296	HIS89	Hydrogen bond	2.95
5	ARG296	GLY95	Hydrogen bond	2.64
6	ARG296	GLY97	Hydrogen bond	2.66
7	ALA297	ILE99	Hydrogen bond	3.17
8	TYR323	HIS222	Hydrogen bond	3.28
9	TYR323	HIS222	Hydrogen bond	3.28
10	TYR326	HIS221	Hydrogen bond	2.77
11	ASP327	TYR100	Hydrogen bond	2.60
12	SER329	ARG102	Hydrogen bond	2.77
13	SER329	ARG102	Hydrogen bond	3.08
14	THR330	TYR100	Hydrogen bond	2.88
15	TYR376	LYS215	Hydrogen bond	2.58

### Molecular dynamics simulations

**Figure 8:**
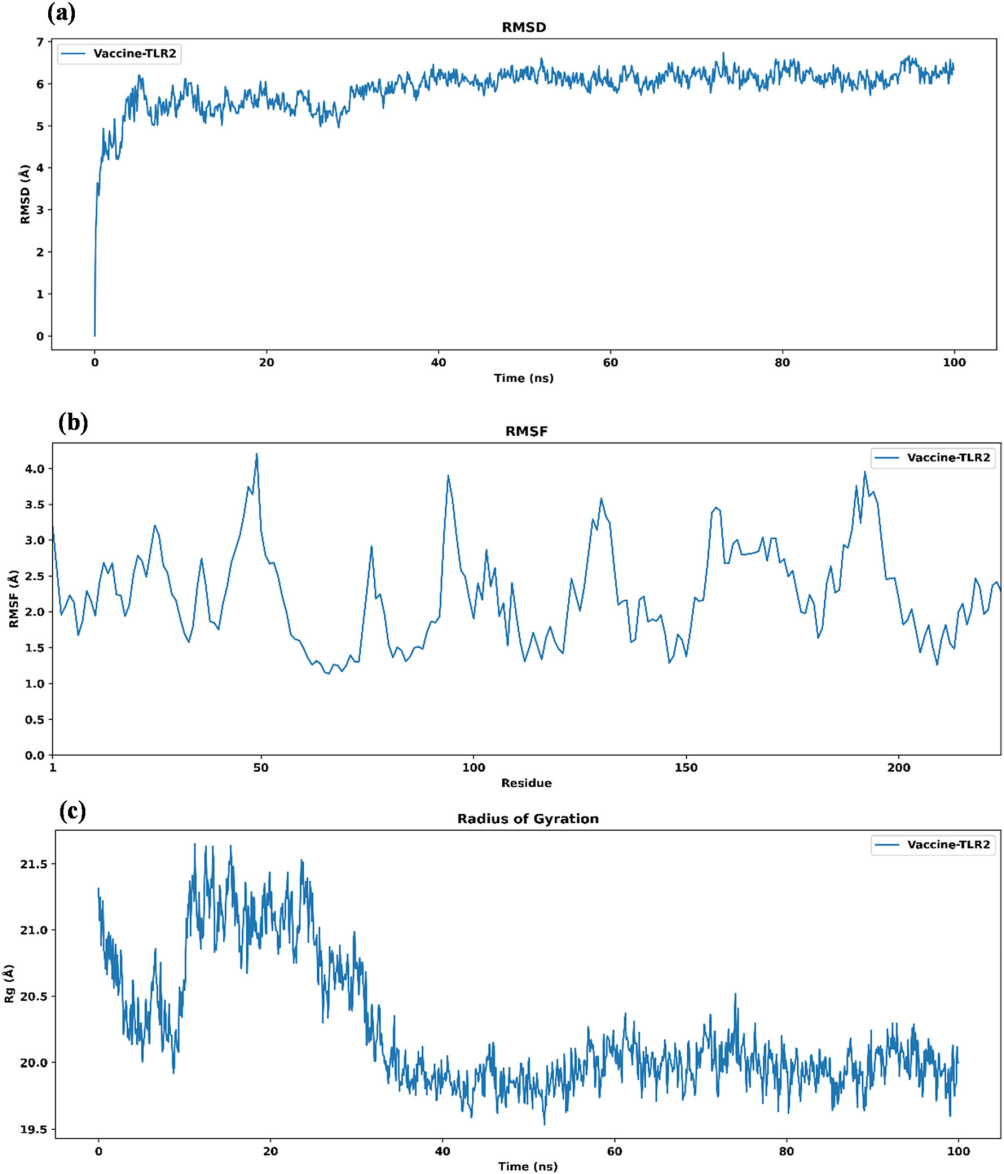
Structural dynamics and stability analysis of the vaccine–TLR2 complex. (a) RMSD graph showing structural stability over a 100 ns simulation, (b) RMSF graph of the vaccine in complex with TLR2, and (c) radius of gyration graph illustrating the compactness of the complex.

Molecular dynamics simulations were conducted for 100 ns to assess the structural stability and conformational behavior of the vaccine-TLR2 complex. The RMSD trajectory (Figure 8a) showed that the complex underwent an initial adjustment phase during the first 5–10 ns, reaching a deviation of approximately 6 Å as the system transitioned from an energy-minimized state to equilibrium. After this equilibration period, RMSD values stabilized between 5.5 Å and 6.5 Å for the remainder of the simulation, indicating that the complex maintained overall structural integrity without significant conformational drifts.

Residue-wise flexibility, represented by RMSF values (Figure 8b), mainly ranged between 1.0 Å and 4.0 Å. Localized peaks were observed around residues 50, 100, 150, and 200, corresponding to loop or surface-exposed regions that naturally exhibit higher mobility. Such moderate flexibility allows antigenic epitopes to remain accessible for immune recognition while preserving the structural stability of the vaccine during interaction with the TLR2 receptor. The relatively low RMSF values across most residues indicate that no significant structural changes occurred during the simulation.

The radius of gyration (Figure 8c) provides insight into the compactness and folding behavior of the vaccine-TLR2 complex. The Rg initially fluctuated between 20.5 Å and 21.5 Å during the first 20 ns, reflecting minor rearrangements as the complex reached equilibrium. After approximately 40 ns, the Rg stabilized around 19.8–20.2 Å, signifying that the system achieved a compact and energetically stable conformation. The stable Rg values toward the end of the simulation further suggest that the complex remained well packed and structurally coherent.

**Figure 9:**
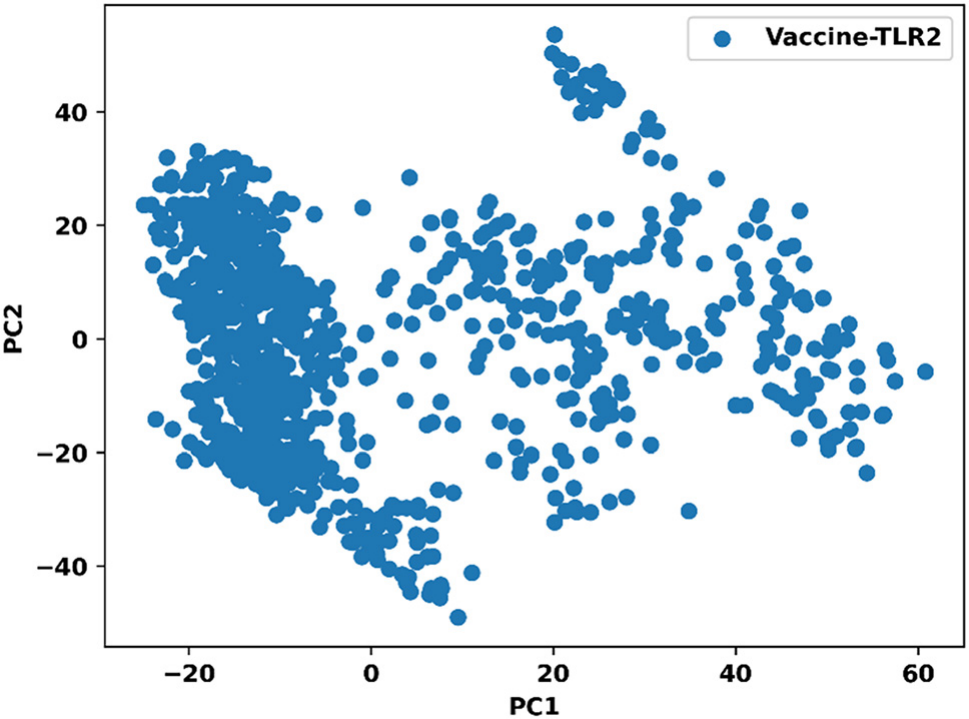
Principal component analysis plot of the vaccine-TLR2 complex showing dominant conformational motions during the 100 ns molecular dynamics simulations.

Principal component analysis was performed to identify the dominant motions within the vaccine-TLR2 system. The clustered distribution of data points indicates that the conformational space sampled during the 100 ns simulation was limited, implying that the system fluctuated within a narrow range of stable conformations (Figure 9). A few scattered points correspond to minor local transitions that reflect natural dynamic flexibility. The prevalence of tightly grouped clusters supports that the vaccine-TLR2 complex achieved a stable conformational ensemble with restricted large-scale motions.

#### Dynamic cross-correlation matrix

**Figure 10:**
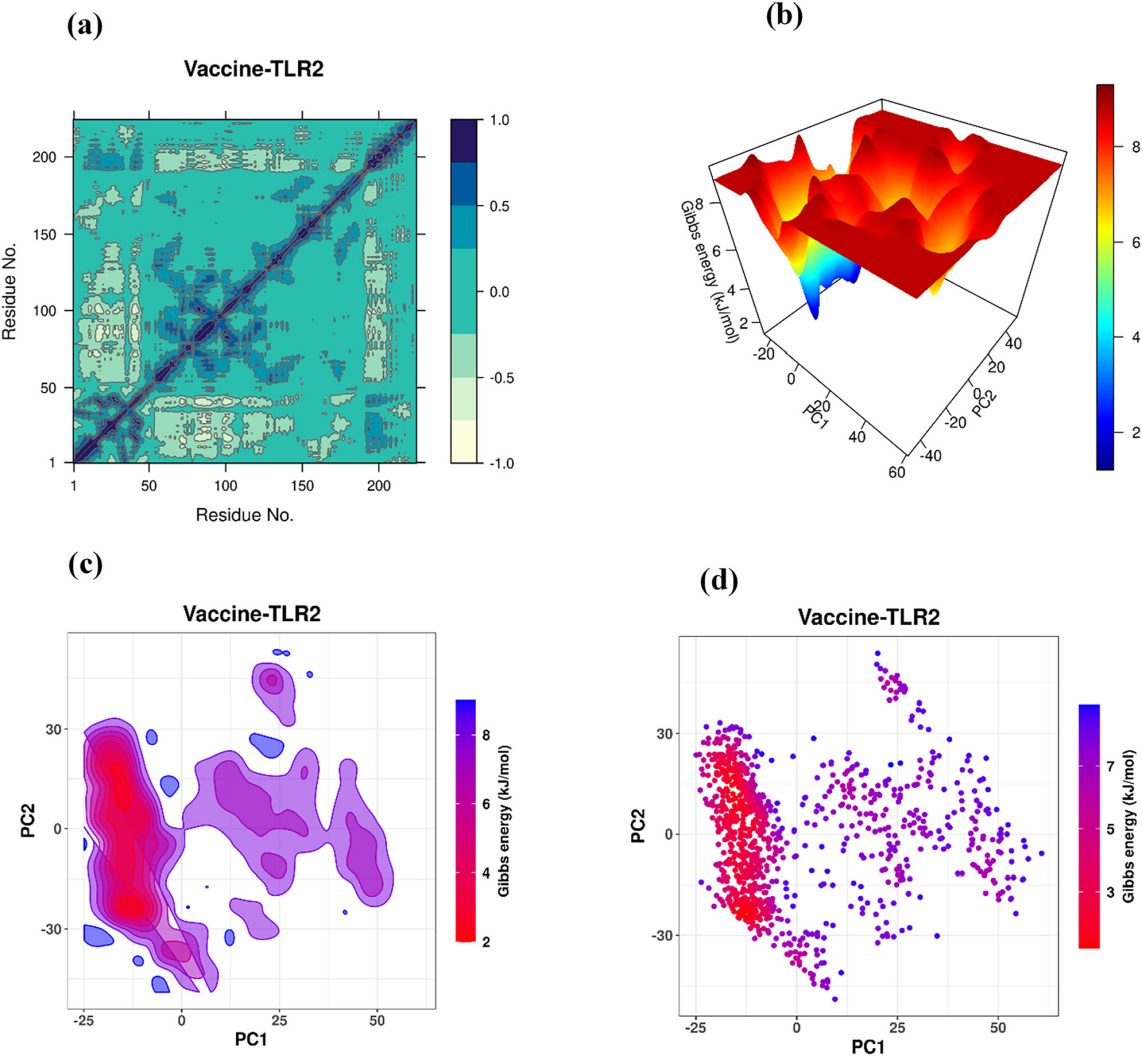
Energy landscape and correlation analysis of the vaccine–TLR2 complex. (a) Dynamic cross-correlation matrix. (b) Three-dimensional free energy landscape (3D FEL) of the vaccine-TLR2 complex. (c) Two-dimensional free energy landscape (2D FEL) contour plot. (d) Gibbs free energy scatter plot.

The DCCM analysis revealed correlated motions among residues within the vaccine-TLR2 complex during the simulation. Positive correlations (dark blue regions) indicated coordinated and stable residue movements, while minor negative correlations in flexible regions represented natural motion variability (Figure 10a). Most residues displayed strong positive correlations (+0.6 to +0.9), suggesting that the vaccine-TLR2 complex maintained coordinated motion and structural consistency throughout the simulation.

#### Free energy landscape

The three-dimensional free energylandscape showed that the Gibbs free energy of the vaccine-TLR2 complex was distributed across low-energy, stable conformations (blue regions). In contrast, higher-energy, less stable states appeared as red regions. The deepest energy wells, observed around 2–3 kJ/mol (Figure 10b), indicate that the complex predominantly occupies thermodynamically favorable conformations. The two-dimensional FEL contour plot (Figure 10c) and Gibbs free energy scatter plot (Figure 10d) further supported these results, with most sampled conformations clustering within the 2–4 kJ/mol range and minor fluctuations observed at 7–8 kJ/mol in flexible loop regions. These findings indicate that the vaccine-TLR2 complex maintained structural compactness and thermodynamic stability throughout the simulation, suggesting a consistent and energetically favorable interaction with the receptor.

### *In silico* cloning

**Figure 11:**
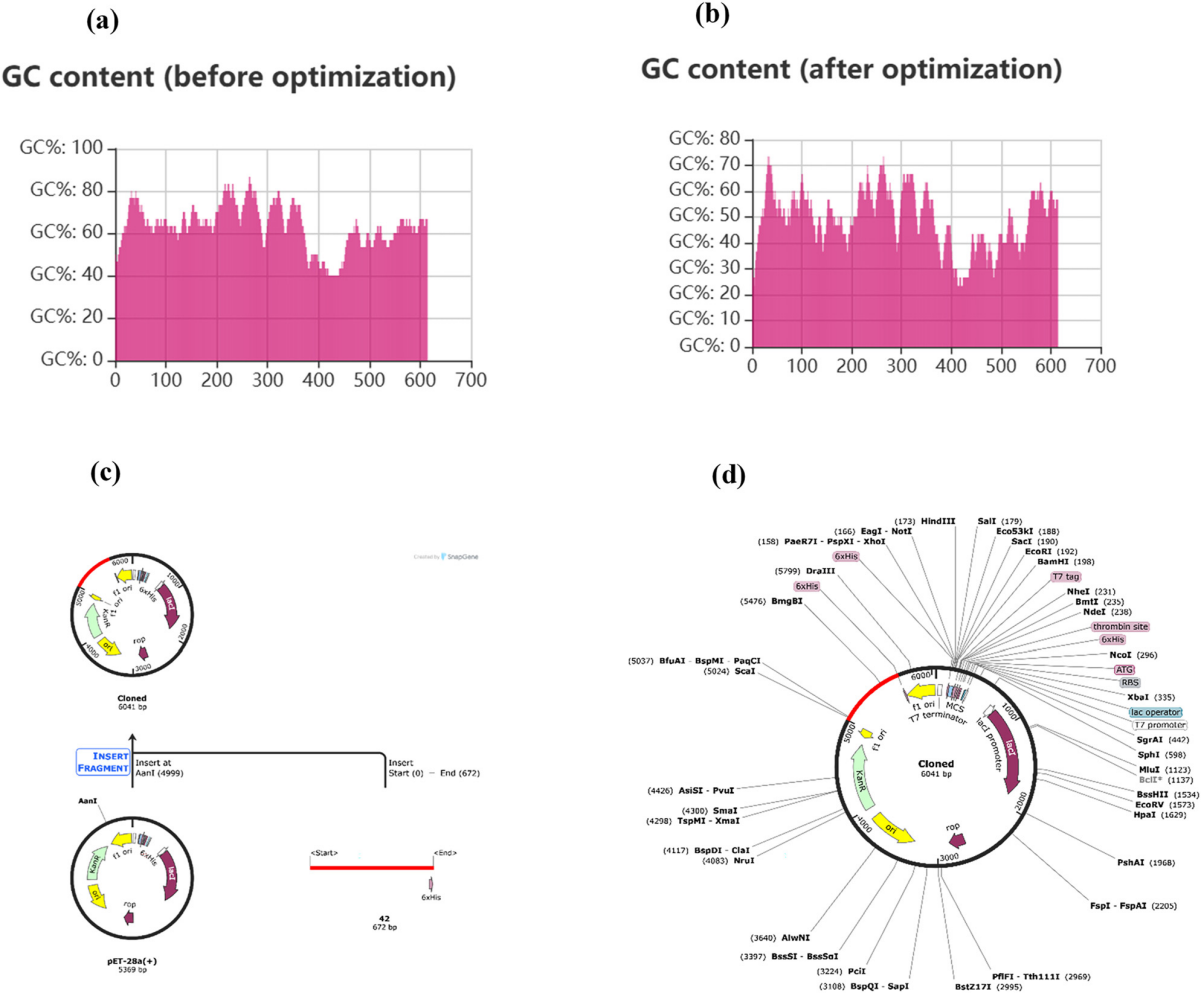
Codon optimization and in silico cloning analysis of the vaccine construct. (a) GC content of the vaccine sequence before codon optimization. (b) GC content after codon optimization. (c) Cloning history showing the insertion of the vaccine gene into the pET-28a(+) vector. (d) Recombinant plasmid map illustrating the key vector components.

Initially, the vaccine protein sequence comprising 224 amino acids was reverse-translated into a nucleotide sequence of 672 base pairs. Codon optimization was performed to enhance expression efficiency in the target host, reducing the GC content from 63.69 % (Figure 11a) to 49.55 % (Figure 11b), which is favorable for *E. coli* expression. The optimized gene sequence was subsequently cloned into the pET-28a(+) vector at the AanI restriction site using SnapGene 8.0.0 (Figure 11c and 11d). The final recombinant plasmid map (Figure 11d) illustrates key expression elements, including the T7 promoter, lac operator, ribosome binding site (RBS), thrombin cleavage site, and His tag, which collectively facilitate efficient expression, purification, and downstream characterization of the vaccine protein.

### Immune simulations

**Figure 12:**
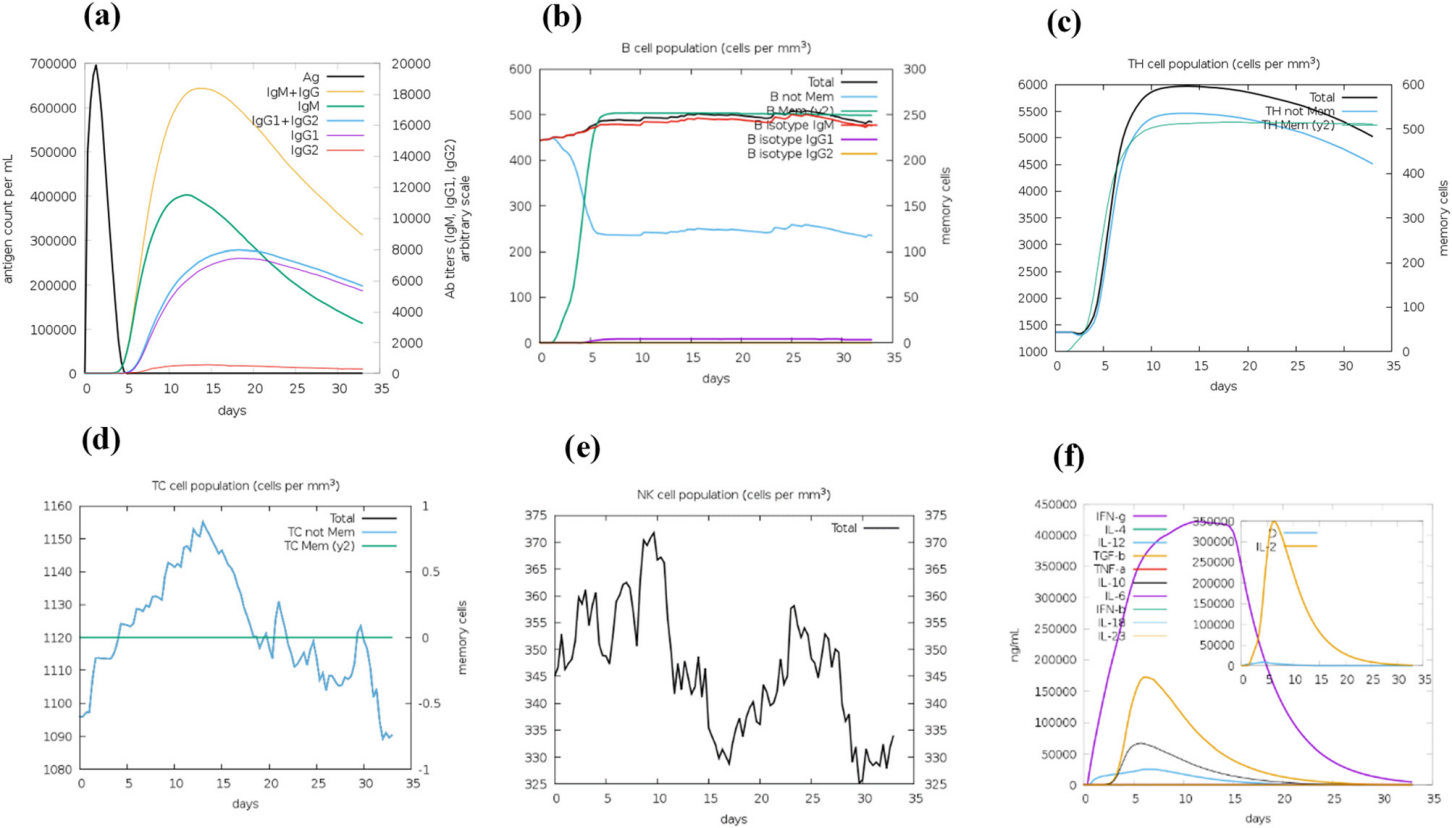
Immune simulation of the designed vaccine. (a) Antigen concentration and immunoglobulin production. (b) B-cell population dynamics. (c) Helper T-cell population. (d) Cytotoxic T-cell population. (e) Natural killer (NK) cell activity. (f) Cytokine production profile.

The immune response simulation performed using the C-ImmSim server predicted that the designed vaccine construct could elicit both innate and adaptive immune responses. Figure 12a shows that the antigen concentration increased to approximately 1,000,000 per mL by day 3 following vaccine administration and subsequently declined due to antibody-mediated neutralization. The IgM response peaked at around 600,000 per mL on day 10, followed by IgG1 and IgG2, which reached approximately 18,000 and 15,000 per mL by day 20, respectively, indicating a possible long-term humoral response. Figure 12b depicts an increase in the B-cell population from about 150 to 280 cells/mm^3^, with memory B cells reaching approximately 100 cells/mm^3^, consistent with antibody production and retention. In Figure 12(c), the T helper (TH) cell population rose from around 1,000 to 5,500 cells/mm^3^ by day 10 and then plateaued, followed by a gradual decline to 500 cells/mm^3^ by day 35, suggesting the presence of memory TH cells. The cytotoxic T-cell (TC) population (Figure 12d) increased from 1,050 to 1,160 cells/mm^3^ by day 15, indicating predicted activation and differentiation into memory subsets. Similarly, the NK cell population (Figure 12e) fluctuated between 335 and 375 cells/mm^3^, with a peak around day 15. Cytokine profiling (Figure 12f) showed elevated levels of IL-2, IFN-γ, and IL-12, which are characteristic of a Th1-type immune response.

While these computational results suggest the potential for a balanced humoral and cellular immune response, they remain theoretical predictions. The C-IMMSIM platform employs algorithmic modeling of immune dynamics and cannot fully replicate biological variability, host–pathogen interactions, or experimental immune kinetics. Therefore, these outputs should be interpreted as indicative rather than conclusive. Experimental verification through *in vitro* and *in vivo* immunological assays will be essential to confirm the predicted antibody titers, cytokine responses, and memory cell formation observed *in silico*.

## Discussion

This study presents an *in silico* multi-epitope vaccine candidate against Tibrovirus Congo built from the viral glycoprotein (G) and nucleoprotein. During the pandemic, there was an accelerated development of SARS-CoV-2 vaccines [54]. Several studies have focused on G protein and nucleoprotein, Spike (S), and nucleocapsid (N) proteins in designing vaccine constructs. In this study, we have utilized similar approaches [55]. The designed construct was predicted to be antigenic, non-allergenic, non-toxic, and structurally stable, with broad global population coverage. To enhance immunogenic potential, a β-defensin-3 adjuvant was positioned at the N-terminus, and standard linkers and a PADRE sequence were incorporated to aid processing and presentation. β-defensins can recruit and activate antigen-presenting cells and support T-helper responses, providing a biologically plausible rationale for inclusion. β-defensins can recruit and activate antigen-presenting cells and support T-helper responses, providing a biologically plausible rationale for inclusion. β-defensins are small antimicrobial peptides synthesized by the innate immune system, and thus, their adaptive immunity plays a crucial role in modulating the innate immune response [56]. These peptides act on antigen-presenting cells (APC) such as dendritic cells (DC) and macrophages, which undergo more effective processing and presentation of the antigens. It has been shown in many reports that β-defensins are natural immunostimulants that cause the T helper (Th1 and Th2) cell responses, which are crucial for robust cellular and humoral immune responses to viral infections [57].

Molecular docking studies were performed to evaluate the potential immune activation of the vaccine construct through interaction with Toll-like receptors (TLR2 and TLR4). TLRs are pattern recognition receptors (PRRs) that detect pathogen-associated molecular patterns (PAMPs) and trigger innate immune signaling pathways [58]. Among them, TLR2 primarily recognizes viral lipopeptides and glycoproteins, making it a key receptor for assessing vaccine-induced immune responses [59]. TLR4, on the other hand, recognizes glycoproteins and lipopolysaccharides and plays an essential role in pro-inflammatory cytokine synthesis and dendritic cell maturation [60].

Docking analysis demonstrated strong binding interactions with both receptors, showing binding energies of −1,665.5 for TLR2 (15 hydrogen bonds) and −1,227.8 for TLR4 (10 hydrogen bonds). These results align with previous findings in SARS-CoV-2 vaccine studies [61], where TLR interactions were linked to enhanced immune activation.

The docking and simulation analyses revealed that the vaccine construct exhibited stronger binding affinity and greater stability with TLR2 compared to TLR4. This difference is biologically significant because TLR2 is more actively involved in recognizing viral glycoproteins and initiating innate immune signaling through the MyD88-dependent pathway. Activation of this pathway leads to NF-κB translocation and the production of pro-inflammatory cytokines such as IL-6, IL-12, and TNF-α [62], 63], which enhance antigen presentation and promote T-cell activation. The relatively lower docking score for TLR4 suggests a cooperative but less dominant role in immune recognition. Molecular dynamics simulations showed that the vaccine-TLR2 complex maintained high structural stability and compactness throughout the simulation, supporting its role in initiating immune activation. The 100 ns MD simulations showed that the vaccine-TLR2 complex remained in dynamic and structural equilibrium, with RMSD values around 6 Å and RMSF fluctuations between 1 Å and 4 Å, indicating stable yet flexible regions favorable for epitope presentation. The Rg profile stabilized around 20.2 Å, confirming compactness and structural integrity. PCA and DCCM analyses highlighted coordinated residue motions and coupled dynamics, while the free energy landscape revealed stable, low-energy conformational states. However, as these findings are computational, *in vitro* receptor activation and cytokine assays will be necessary to validate their biological relevance.

The immune simulation analysis provided additional insights into the potential immunological performance of the vaccine construct. The observed peak of IgM levels followed by a substantial and sustained increase in IgG1 and IgG2 antibodies reflects a typical primary and secondary immune response pattern, similar to that seen in effective viral vaccines. The early IgM surge indicates initial antigen recognition, while the elevated IgG titers signify immunological memory and long-term protection. Additionally, the marked production of IL-2 and IFN-γ corresponds to the activation of Th1-type cellular immunity, which enhances cytotoxic T-cell activity and promotes viral clearance [64]. This simulated immune profile is comparable to responses induced by other multi-epitope and peptide-based vaccine models, suggesting that the designed construct may trigger balanced humoral and cellular immune responses [65], 66]. Nonetheless, these predictions require experimental validation to confirm cytokine induction and antibody kinetics under biological conditions.

Despite the promising computational outcomes, this study has several limitations that must be acknowledged. The entire analysis was conducted using *in silico* methods, which, although powerful for early vaccine design, cannot fully replicate the complexity of immune responses in biological systems. Computational predictions of docking, molecular dynamics, and immune simulations are based on theoretical models and therefore require experimental validation to confirm their accuracy and biological relevance. To address these limitations, future work will focus on the experimental validation of the designed multi-epitope vaccine construct through a structured series of studies. The construct will first be synthesized, cloned into the pET-28a(+) vector, and expressed recombinantly in *E. coli*. This will be followed by purification and characterization using SDS-PAGE and Western blotting [67]. *In vitro* immunological assays, including cytokine profiling (IL-2, IFN-γ, TNF-α) and antigen-antibody binding analysis via ELISA, will be performed using peripheral blood mononuclear cells to assess immunogenicity and safety [68]. Subsequently, *in vivo* testing in BALB/c mice will be conducted to evaluate antibody titers, T-cell proliferation, cytokine responses, and safety parameters, followed by challenge studies with recombinant Tibrovirus antigens to determine protective efficacy [69]. This stepwise experimental plan provides a clear pathway to validate the computational predictions, bridge the gap between *in silico* modeling and biological systems, and advance the vaccine candidate toward preclinical development.

## Conclusions

This study presents an *in silico* design of a multi-epitope vaccine candidate against Tibrovirus Congo, targeting its glycoprotein (G) and nucleoprotein. Through immunoinformatics and structural vaccinology approaches, the vaccine construct was predicted to be highly antigenic, non-allergenic, non-toxic, and physicochemically stable, with an estimated global population coverage of 91.81 %. Molecular docking and dynamics simulations indicated stable, high-affinity interactions with TLR2, while immune simulation analysis predicted balanced humoral and cellular immune responses with cytokine secretion and memory cell formation. Incorporation of an adjuvant, disulfide engineering, and codon optimization further enhanced the predicted immunogenicity and expression potential. Overall, the designed construct represents a promising candidate for *in vitro* and *in vivo* validation to support vaccine development against Tibrovirus Congo and related viral pathogens.
